# 肺部典型类癌全身多处转移1例

**DOI:** 10.3779/j.issn.1009-3419.2010.05.32

**Published:** 2010-05-20

**Authors:** 琦 桂, 澄澄 徐, 世英 于

**Affiliations:** 1 430030 武汉，华中科技大学附属同济医院肿瘤科 Department of Oncology, Affiliated Tongji Hospital of Huazhong University of Science and Technology, Wuhan 430030, China; 2 430030 武汉，华中科技大学附属同济医院普胸外科 Department of Thoracic Surgery, Affiliated Tongji Hospital of Huazhong University of Science and Technology, Wuhan 430030, China

## 1 病史简介

患者女性，36岁，2004年8月体检发现右肺近肺门处一小结节（图 1），2005年4月患者在当地行手术切除，术后诊断为右肺中低分化鳞癌pT2N0M0，行紫杉醇加顺铂方案化疗6个周期，后自服中药治疗，定期复查未见明显病变进展。2009年4月发现双乳包块，于当地行包块切除术，术后病检送至多家医院会诊，结果均为皮肤汗腺来源肿瘤。2009年7月，出现上腹部不适及颜面部皮疹，并出现右锁骨上及耳后下质硬肿大淋巴结，腹部CT示肝占位性病变（图 1）。患者既往无吸烟病史，患者母亲于1年前诊断为肺鳞癌骨转移。

**1:**
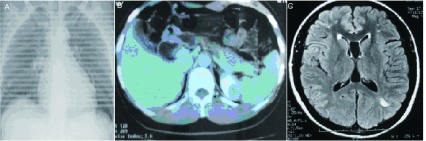
肿瘤原发及转移部位影像学资料。A：CT示右肺门处一3 cm×4 cm团块影；B：腹部转移灶；C：脑部转移灶 Primary tumor and metastatic sites radiographic imagings. A: CT showing a 3 cm×4 cm clumps shadow on the hilum of lung; B: Abdominal metastasis; C: Brain metastasis

## 2 辅助检查

入我院后完善相关检查，血常规、生化常规、肿瘤标志物CEA、NSE、CA15-3均未见异常。骨扫描未见明显异常。行头部磁共振发现脑多发占位病变，考虑为脑转移（图 1）。会诊外院腹部CT示右肝前下段结节，多为转移性肿瘤，双肾多个小占位，考虑错构瘤。

## 3 病理

2009年4月乳腺包块外院病检结果为（乳腺）皮肤良性肿瘤，倾向透明细胞汗腺瘤，免疫组化：CK少数腺体（+），Calponin（-），S-100（-），P63（-），Ki67 < 2%（+），SMA部分（+），CK5/6（-），ER（-）。送检肺及乳腺手术后病检切片于我院病理科会诊，病检结果（右肺）典型类癌伴（乳腺）转移，免疫组化：Syn（+）CD56（+）CgA（+）（“肺”及“乳腺”两处免疫组化结果相同）。为进一步明确诊断，取右耳后下肿块活检，病检结果为（右腮腺区）转移性类癌，结合临床，考虑来源于肺，免疫组化：Syn（+）CD56（+）CgA（+），PCK弱（+），CK5/6（-），CK7（-），TTF-1（-），GCDFP-15（-），P63（-），SMA（-）（图 2）。

**2:**
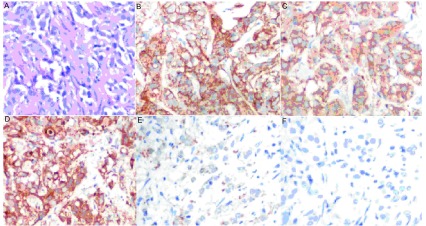
右耳后活检光镜及免疫组化图片。A：右耳后下肿块活检病理切片光镜（HE，×200）；B：右耳后下肿块活检免疫组化CD56（+）（×100）；C：右耳后下肿块活检免疫组化Syn（+）（×100）；D：右耳后下肿块活检免疫组化CgA（+）（×100）；E：右耳后下肿块活检免疫组化PCK弱（+）（×100）；F：右耳后下肿块活检免疫组化CK7（-）（×100） Light microscopy and immunohistochemical pictures of tumor biopsy under the right ear. A: Slices of tumor biopsy under the right ear, light microscope (HE, ×200); B: Immunohistochemistry of tumor biopsy under the right ear, CD56 (+) (×100); C: Immunohistochemistry of tumor biopsy under the right ear, Syn (+) (×100); D: Immunohistochemistry of tumor biopsy under the right ear, CgA (+) (×100); E: Immunohistochemistry of tumor biopsy under the right ear, PCK week (+) (×100); F: Immunohistochemistry of tumor biopsy under the right ear, CK7 (-) (×100)

## 4 诊断

右肺典型类癌治疗后复发（双乳、肝、脑、锁骨上及耳后淋巴结转移）。

## 5 分析讨论

肺类癌较肺内其它恶性肿瘤及肠道类癌少见，国际癌症生存流行病学机构（Surveillance Epidemiology and End Results data, SEER）的数据^[1]^显示肺类癌占全部肺原发恶性肿瘤的1.2%，其中肺典型类癌占肺类癌的80%-90%^[2]^。肺典型类癌通常为靠近中心的单发病灶，分化较好^[3]^，病情进展缓慢，远处转移少见，预后较好，5年生存率为87%-89%^[4]^。由于肺典型类癌缺乏典型临床症状，激素相关性症状较肠类癌少见，不到5%的肺神经内分泌肿瘤患者出现激素相关症状，如类癌综合征、Cushing综合征、肢端肥大症及抗利尿激素分泌异常综合征^[5]^，且病理检查中细胞形态不典型，故临床上较易误诊。仅3%的肺典型类癌患者发生远处转移，部位为肝脏、骨骼、中枢神经系统、皮肤及乳腺^[5]^。

病理评估：肺类癌与其它原发于肺的神经内分泌肿瘤的组织学鉴别主要根据细胞增殖标志物Ki67的百分比以及一些肽类和胺类标志物，包括CgA、NSE、血清素、Syn和ACTH。而进一步区分肺典型和不典型类癌主要根据核分裂数目及有无坏死（WHO对肺典型类癌的诊断标准为具有类癌形态的肿瘤，10HPH下 < 2核分裂相/2mm^2^，且无坏死结构）。本例患者2005年4月肺部肿块病理切片光镜下仅可见上皮细胞团，无角化珠、细胞间桥等鳞癌特征，故鳞状细胞癌的诊断有误，而皮脂汗腺来源肿瘤与神经内分泌肿瘤光镜下难以鉴别，需加做上述标志物的免疫组化检测。

后续治疗：对于肺类癌患者，目前主要的和唯一的根治性治疗仍然是完整的手术切除。肺类癌普遍对放疗抵抗，对于生长抑素受体过度表达的患者，可考虑90-Yttrium-DOTA放射性核素的肽受体放疗，发表于2009年美国临床肿瘤学会（American Society of Clinical Oncology, ASCO）会议的一项研究早期数据肯定了90-Yttrium-DOTA-肽受体放疗治疗进展期神经内分泌肿瘤疗效及安全性^[6]^。化疗对肺类癌的作用有限（有效率仅为20%-30%），且有效维持时间短，目前对不可切除病灶尚无证据充足的治疗方案^[7]^。化疗药通常选用铂类和链脲菌素类。虽然肺类癌高表达生长抑素受体，但生长抑素类似物的治疗作用仍然有限，无远处转移患者中仅对伴有类癌综合征和Cushing综合征的患者改善症状疗效明确。2009年ASCO发表了长效奥曲肽治疗肠道转移性神经内分泌肿瘤Ⅲ期研究结果，其显著延长了中位肿瘤进展时间及降低肿瘤进展风险^[8]^。但对肠道外原发的神经内分泌肿瘤临床试验正在进行中，目前尚无确切结果。此外，关于长效奥曲肽联合everolimus（m-Tor抑制剂）的研究RADIANT正在进行，有较好前景^[9]^。

对于本例患者，我们将化疗基因送检并检测，以筛选敏感安全化疗方案，同时遵照美国国家综合癌症网（National Comprehensive Cancer Network, NCCN）2009年指南，行短效奥曲肽治疗（0.15 mg, tid），充分评估患者安全性及耐受性后，行长效奥曲肽治疗。1个月后复查评估疗效：肝脏病灶直径由2.2 cm增至2.8 cm，脑部病灶几乎无改变，综合评价疗效为稳定。

## References

[b1] 1The US National Cancer Institute. Surveillance Epidemiology and End Results (SEER) data base, 1973-2004, http://seer.cancer.gov/2007.

[2] Fink G, Krelbaum T, Yellin A (2001). Pulmonary carcinoid: presentation, diagnosis, and outcome in 142 cases in Israel and review of 640 cases from the literature. Chest.

[3] Rosado de Christenson ML, Abbott GF, Kirejczyk WM (1999). Thoracic carcinoids: radiologic–pathologic correlation. Radiographics.

[4] Skuladottir H, Hirsch FR, Hansen HH (2002). Pulmonary neuroendocrine tumors: incidence and prognosis of histological subtypes. A population-based study in Denmark. Lung Cancer.

[5] Gustafsson BI, Kidd M, Chan A (2008). Bronchopulmonary neuroendocrine tumors. Cancer..

[b6] 6Toumpanakis C, Quigley A, Srirajaskanthan, *et al*. 90-Yttrium-DOTA-octreotate for the treatment of advanced neuroendocrine tumors. J Clin Oncol, 2009 ASCO Annual Meeting Proceedings (Post-Meeting Edition), 2009, 27(15s): May 20 Supplement.

[7] Beasley MB, Thunnissen FB, Brambilla E (2000). Pulmonary atypical carcinoid: predictors of survival in 106 cases. Hum Pathol.

[8] Rinke A, Müller HH, Schade-Brittinger C (2009). Placebo-controlled, doubleblind, prospective, randomized study of the effect of octreotide LAR in the control of tumor growth in patients with metastatic neuroendocrine midgut tumors: A report from the PROMID study group. J Clin Oncol.

[9] Yuan R, Kay A, Berg WJ (2009). Targeting tumorigenesis: development and use of mTOR inhibitors in cancer therapy. J Hematol Oncol.

